# Dopamine D2 receptor gene Taq 1A polymorphism: genetic architecture in Indian population and comparison to global populations

**DOI:** 10.3389/fgene.2025.1610364

**Published:** 2025-07-14

**Authors:** Amrita Chaudhary, Pradeep Kumar, Bechan Sharma, Vandana Rai

**Affiliations:** ^1^ Human Molecular Genetics Laboratory, Department of Biotechnology, Veer Bahadur Singh Purvanchal University, Jaunpur, India; ^2^ Department of Biochemistry, University of Allahabad, Prayagraj, India

**Keywords:** dopamine receptor D2 (DRD2), dopamine, Taq1A, polymorphism, PCR-RFLP, psychiatric disorder, India

## Abstract

**Objective:**

The Dopamine receptor D2 (DRD2) gene has been investigated as a candidate gene in several psychiatric and neurological disorders involving dopaminergic systems. Multiple polymorphisms have been reported in the DRD2 gene, where the DRD2 Taq1A is most widely studied and is reported to contribute to the development of several diseases/disorders. The objective was to study the DRD2 Taq1A polymorphism in the Indian population and compare it with the reported global frequency.

**Methods:**

The DRD2 Taq1A polymorphism was genotyped using PCR-RFLP from 400 unrelated human blood samples. In addition, a literature search on the DRD2 Taq1A polymorphism has also been conducted from 1990 to 2025. All the data obtained was grouped according to the continent as a unit to get the distribution information of the DRD2 Taq1A genotypes and alleles in healthy populations of all six continents. This was accomplished for the comparison of frequency obtained in this study with the overall geographical distribution of the DRD2 Taq1A allele reported from other parts of the world.

**Results:**

In the total 400 samples analyzed, the TT genotype and T allele frequencies were 15% and 43%, respectively. Data from 136 studies from different continents were extracted and compared with the present study. The highest T allele frequency was observed in the Asia (0.35), followed by South America (0.33) and the lowest frequencies occur in Europe and Australia (0.19). Pattern of frequencies represented by the Indians is consistent to Asia and close to South America. The result show a high frequency of CT genotype and T allele in the study population, closely resembling the patterns observed in Mexicans. This study highlights the genetic diversity within Indian subpopulations and underscores the need for cautious interpretation of population genetic data.

**Conclusion:**

The present study observed a T allele frequency of 43%, comparable with the Asian population. In the comparison study, the T allele frequency in Global, Asian, Indian, and present studies was calculated as 26%, 35%, 33%, and 43%, respectively. This geographical gradient is clinically important in determination of the risk assessment which might be included in prevention strategies for psychiatric disorders.

## Introduction

Dopamine is one of the major neurotransmitters produced in the neuronal terminals by two consecutive steps of hydroxylation and decarboxylation of the amino acid tyrosine. All the physiological functions guided by dopamine are mediated by the dopamine receptors. Dopamine receptors belong to the superfamily of seven-pass transmembrane G protein-coupled receptors (GPCR). Dopamine receptors are classified into two subfamilies -the D1-like subfamily (dopamine receptor D1 and D5), and the D2-like subfamily (dopamine receptor D2, D3, and D4) according to their capability to stimulate the adenylyl cyclase. D1-like receptors stimulate the adenylyl cyclase and hence increase the production of cyclic AMP (cAMP) whereas, D2 like receptors inhibit the production of cAMP (Lee and Wong, 2009; Beaulieu et al., 2015; Martel and Gatti McArthur, 2020; Sarkar et al., 2024). The dopamine system regulates a diverse set of neural systems including movement, locomotion, reward, cognitive process, and endocrines regulation. Therefore, any genetic variations in these receptor genes alter the dopaminergic neurotransmission in the brain and increase the susceptibility to various neuropsychiatric disorders like autism spectrum disorder (ASD) (dopamine receptor D1), schizophrenia (dopamine receptor D3), Parkinson’s disease (dopamine receptor D3 and dopamine receptor D4), and paranoid schizophrenia (dopamine receptor D5) (Kim et al., 2009; Zhao et al., 2014; Marriggio et al., 2021; Dipanwita et al., 2022; Sofronov et al., 2022; Morozova et al., 2024; Sarkar et al., 2024).

Dopamine receptor D2 is widely investigated due to its pivotal role in the regulation of dopamine action which is mostly expressed in the striatum. Alterations in the dopamine receptor expression, localization and function affects the reward pathways and responses associated with it (Vallone et al., 2000; De Mei et al., 2009; Lee and Wong, 2009; Blum et al., 2023). It imposes significant implications in several neuropsychiatric disorders such as schizophrenia (Alfimova et al., 2019), ASD (Sukasem et al., 2016), attention deficit hyperactivity disorder (ADHD) (Paclt et al., 2010), Parkinson’s disease (Hassan et al., 2016), post-traumatic stress disorder (PTSD) (Hemmings et al., 2013), bipolar disorder (Wang et al., 2014), and obsessive-compulsive disorder (OCD) (Lochner et al., 2016).

The dopamine receptor D2 (DRD2) gene is located on chromosome 11q22-23 and extends over 270 kilobases, with eight exons. The gene encodes two isoforms a short D2-short (D2S) isoform of 414 amino acids and a long D2-long (D2L) isoform of 443 amino acids (He et al., 2013; Jasiewicz et al., 2014). The two isoforms are generated by the alternative splicing of 87bp long exon 6. D2L is expressed mostly post-synaptically, while D2S is mostly localized at the presynaptic nerve terminal (Luykx et al., 2017; Lira and Ahammad, 2021).

**FIGURE 1:**
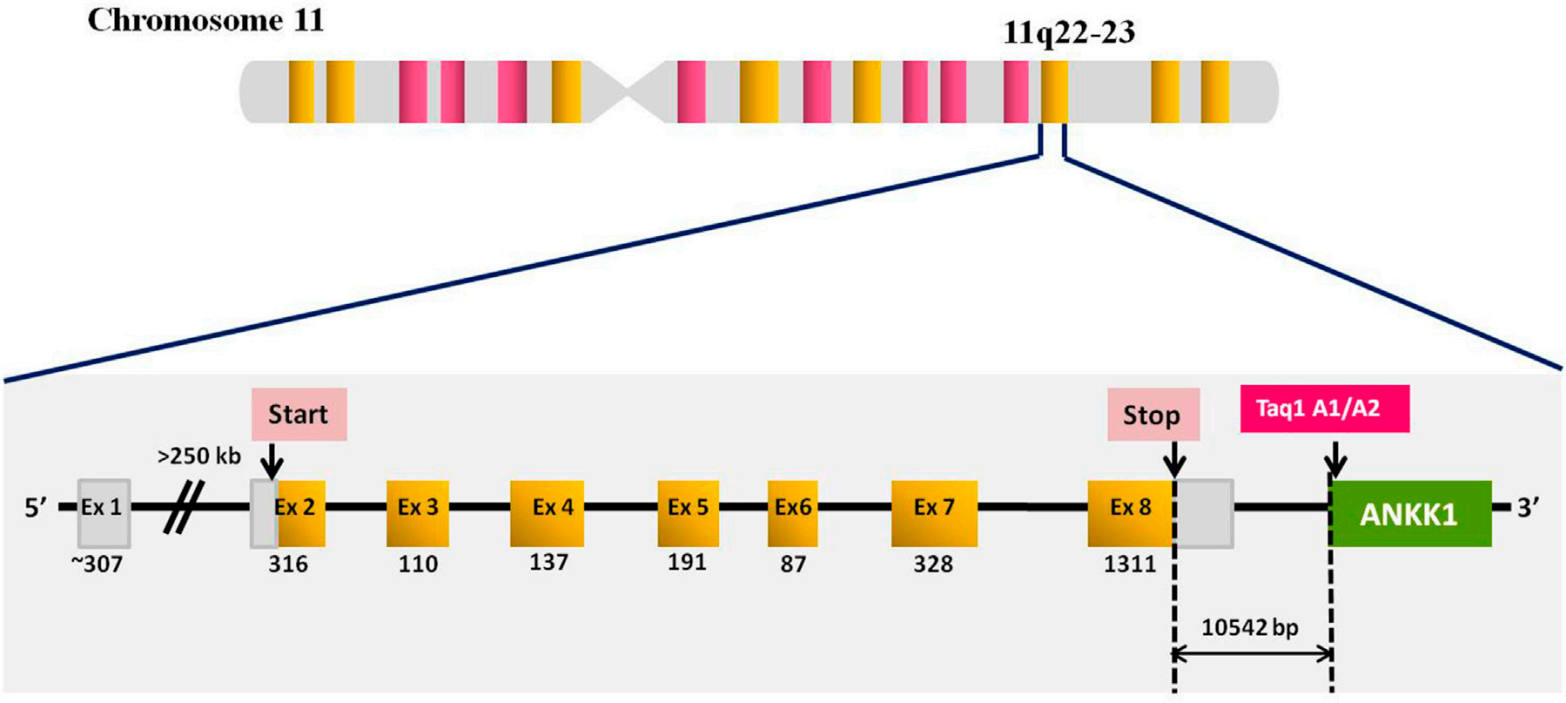
Diagrammatic representation of DRD2 gene structure and location of DRD2 Taq1A polymorphism. DRD2 locus with exon numbers (in boxes), and exon sizes (below each exon boxes in bp). Yellow blocks represent the coding region (exon 2-8) covering 13 925 bp of the gene and white blocks represent the 5' UTR and 3'UTR.

A large number of polymorphisms are reported in the DRD2 gene, out of which the DRD2 Taq1A polymorphism is extensively investigated. The C>T single nucleotide polymorphism is located ∼10 kb downstream of the DRD2 gene in the eighth exon of the neighboring gene, ankyrin repeat and kinase dopamine-containing 1 (ANKK1) known as DRD2/ANKK1 or DRD2 Taq1A (Glu713Lys, C/A2→T/A1, rs1800497) (Figure 1). Although, Taq1A polymorphism is not located in the DRD2 gene, however, it affects the expression, availability, and affinity of the DRD2 receptors. This functional polymorphism affects the 12 ankyrin repeat domains of the ANKK1 protein. Therefore, it is likely that this SNP affects ANKK1 function by changing its ability to bind to other proteins (Ponce et al., 2009).

It is well documented that the minor T allele results in the reduced expression of the dopamine receptor in the striatum (Neville et al., 2004; Kaminskaite et al., 2021). Reduced expression of dopamine receptors, results in the dysfunction of the dopaminergic neurotransmission (Panduro et al., 2017; Kaminskaite et al., 2021). This polymorphism has been reported as a risk factor for several psychiatric as well as neurological disorders (Kumudini et al., 2013; Mayer et al., 2015; Cai et al., 2015; Sukasem et al., 2016; Alfimova et al., 2019).

The DRD2 taq1A polymorphism is a key modulator of dopamine receptor density that influences the dopaminergic signaling, thereof play crucial role in the cognition, motivation, reward behaviors, and emotions (Kaminskaite et al., 2021). It is of clinical importance in the Indian population since India is facing a growing burden of psychiatric disorder, and its diverse genetic makeup shaped by regional variation, caste system, endogamy and migration history (Sinha et al., 2015; India State-Level Disease Burden Initiative Mental Disorders Collaborators, 2020). Study of DRD2 Taq1A polymorphism in Indian population provides insight into differential susceptibility to psychiatric disorders, and possible pharmacogenetic responses. These data would be helpful in precision medicine and appropriate mental health interventions.

The frequency of DRD2 Taq1A polymorphism is studied in several ethnicities worldwide. However, the frequency of DRD2 Taq1A polymorphism is relatively unexplored in the Indian population (natives of Eastern Uttar Pradesh). Therefore, the present study aimed to identify the frequency of this gene polymorphism in the Indian population and compare the results with the global frequencies reported from various studies.

## Materials and methods

### Sample collection, DNA isolation and quantification

The 3 mL blood samples were collected in EDTA-coated vials from 400 random unrelated healthy subjects (314 males and 86 females; age: (mean ± SD) = (37.7 ± 15.67) from Eastern Uttar Pradesh population of India. Inclusion Criteria: 1. Random healthy individuals, without any family history of psychiatric disorders, cancer, or other pathological conditions. 2. They must be residents of Eastern Uttar Pradesh, India. 3. Individuals willing to participate. Approval for this study was granted by the Institutional Ethics Committee (IEC) of VBS Purvanchal University, Jaunpur. Genomic DNA was extracted from each collected blood sample according to the protocol of Bartlett and White, (2003), and informed written consent was obtained from each participant prior to blood sample collection. DNA samples were checked for both the quantity and the quality using spectrophotometer (Microprocessor UV/VIS Double beam Spectrophotometer, Model: LI-2700).

### Genotyping

DRD2 Taq1A genotyping was done by the polymerase chain reaction (PCR) by using gene-specific primers and restriction digestion by the restriction enzyme Taq1 (catalogue no: 0100700021730, GeNei) according to the protocol of Grandy et al. (1993). A 310 bp fragment containing the polymorphic region 10 kb downstream from the DRD2 gene was amplified by using the set of forward (F:5′- CCG​TCG​ACG​GCT​GGC​CAA​GTT​GTC​TA- 3′) and reverse primers (R:5'- CCG​TCG​ACC​CTT​CCT​GAG​TGT​CAT​CA-3′). Primers used for this study was taken from the protocols of Grandy et al. (1993) however, it also have been validated by primer designing tool: Primer3web version 4.1.0. The PCR amplification was carried out in the 15 μL reaction mixture containing 100–150 ng of DNA template, 1X buffer, 1 U of *Taq* DNA polymerase (Merck, GeNei), 250 µM dNTP mix (Merck, GeNei), 4 pM of primers (Eurofins genomics) and 0.1% triton X-100. Initial denaturation at 94° for 5 min followed by the 30 cycles involving denaturation at 94 °C for 1 min, annealing at 62°C for 1 min extension at 72°C for 1.30 min, and a 10-min elongation step at 72°C at the end of the cycle. Amplicons were digested by the restriction enzyme Taq1 (GeNei) at 65 °C for 3 h to find out the genotypes. The A1 (T) alleles remained intact (310 bp fragment), whereas C alleles (wild allele) were cut into two fragments of 180 bp and 130 bp.

### Statistical analysis

Gene counting method is used for the calculation of the genotypic and allelic numbers.

The DRD2 Taq1 A (rs1800497) genotype distribution was tested according to the Hardy-Weinberg equilibrium (HWE) with the HWE software https://wpcalc.com/en/medical/equilibrium-hardy-weinberg/. (accessed on 3 June 2024).

Inbreeding coefficient was calculated using the formula:

$$F\;\left(\text{is}\right)=P\;\left(\text{AA}\right)/\;P\;\left(A\right)+P\;\left(\text{aa}\right)/\;P\;\left(a\right)-1$$

Where, P = frequency, A = major allele, a = minor allele, AA = homozygous major allele, aa = homozygous minor allele (Sen and Burmeister, 2008).

Prevalence was calculated from the number of alleles/genotypes and total alleles/sample size with the corresponding 95% confidence interval (CI). The analysis was based on the geographical area, i.e., the continents from where the study was included (North America, South America, Europe, Africa, Asia, and Australia). Every p-value has a two-tailed significance level of less than 0.05. By combining the frequency of DRD2 Taq1A in different countries of several continents, a Global frequency map was created by an application named mapchart.net.

### Document retrieval

Literature on the DRD2 Taq1A polymorphism reported in several studies from various countries from 1990 to 2025 was retrieved from PUBMED, Springer, Science Direct, and Google Scholar databases. The combined review of references cited in various research articles used keywords such as DRD2, dopamine receptor D2, Taq1 A, polymorphism, and psychiatric disorders. Inclusion criteria: 1. Studies published from 1990 to 2025, 2. Subjects included belong to healthy population only, 3. Reports on the frequency of DRD2 Taq1A polymorphism, case-control studies in association with various psychiatric disorders. Exclusion criteria: incomplete data, book chapters, study design such as cohort studies, study including only case and non-original studies such as reviews and meta-analysis.

### Data extraction

Subsequent data were taken from every eligible study, including the name of the first author, the year of the publication, the journal name, the country name, the population/ethnicity, the allele, and the genotype number. When samples from several countries or different caste groups were used in a study, data was extracted independently for each of those nations, races, or castes.

PubMed, Springer links were searched for articles in which authors analyzed DRD2 Taq1A polymorphism to understand the pattern of distribution DRD2 Taq1A polymorphism and to compare with the present study results. A total 136 articles were published from different ethnicities/countries/continents. For convenience, we compared, summarized, and grouped the articles continent-wise. Continent-wise T allele frequency was calculated from the previously published data. Among all 136 articles, there are 24,037 research participants from different ethnic population.

## Results and discussion

**FIGURE 2:**
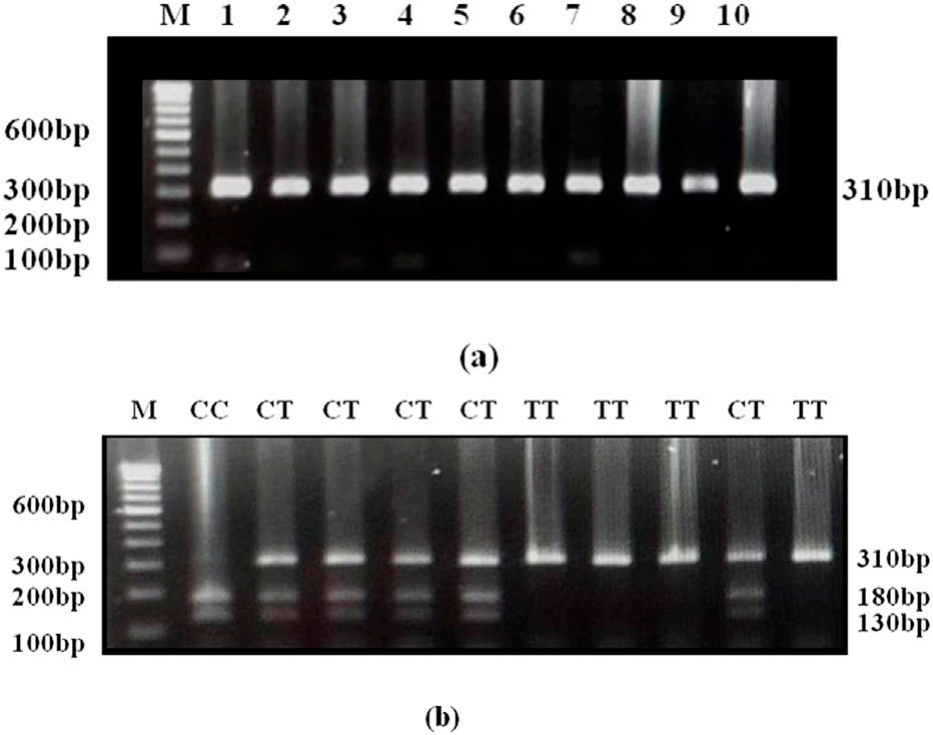
**(a)** Gel picture showing amplicon of 310bp of DRD2 Taql. **(b)** Gel picture of Taql digested DRD2 PCR products. Homozygous wild (CC): 180 and 130bp; Heterozygous (CT): 310, 180 and 130bp; Homozygous mutant (TT): 310bp (remains intact).

The investigation for DRD2 Taq1A C/A2>T/A1 genotype in the population under study depends on the cleavage profile of the restriction enzyme *Taq* 1. Figure 2a illustrates the gel picture showing the amplicon of size 310 bp. The amplicon was digested with Taq1 restriction enzyme. The C allele was digested into two fragments (180 and 130 bp). The C to T substitution abolishes the restriction site and therefore, the amplicon remains intact in the case of T allele Figure 2b.

A significant HW departure (p = 0.014) was found in the studied population, indicating a remarkable heterozygous excess (F (is) = -0.122). The F (is) is a useful metric for measuring the deviation from HWE (HWD). A positive value suggest an excess of homozygotes while a negative value manifest an excess of heterozygotes or deficit of homozygotes (Sen and Burmeister, 2008). Among the 400 unrelated DNA samples were genotyped for the DRD2 Taq1A (rs1800497) polymorphism, the observed genotype frequencies were as follows: A2A2 = 118 (29.5%), A1A2 = 220 (55.0%), and A1A1 = 62 (15.5%). The allele frequencies were A2 = 0.57 and A1 = 0.43. The expected genotype frequencies under HWE were A2A2 = 129.96, A1A2 = 196.08, and A1A1 = 73.96. A significant deviation from HWE was observed from the expected equilibrium (Table 1), which is characterized by an excess of heterozygotes (observed = 220 vs expected = 196.08).

**TABLE 1:** Genotype and allele frequency in India and present study.

India					Genotypes			Allele distribution				HWE
S. No.	Study	Country	Ethnicity	Sample size	CC	CT	TT	A2/C		A1/T		
					No.	No.	No.	No.	Freq	No	Freq	*p-value*
1	Shaikh et al. (2001)	India (South)	Asian	53	15	31	7	61	0.58	45	0.42	0.15
2	Juyal et al. (2006)	India (North)	Asian	130	65	43	22	173	0.67	87	0.33	0.003
3	Vijayan et al. (2007)	India (South)	Asian	194	88	77	29	253	0.65	135	0.35	0.08
4	Singh et al. (2008)	India (North)	Asian	100	49	37	14	135	0.68	65	0.33	0.117
5	Bhaskar et al. (2010)	India (South)	Asian	115	35	51	29	121	0.53	109	0.47	0.23
6	Prasad et al. (2010)	India (North)	Asian	60	38	18	4	94	0.78	26	0.22	0.36
7	Kumudini et al. (2013)	India (South)	Asian	186	91	74	21	256	0.69	116	0.31	0.31
8	Singh et al. (2013)	India (North)	Asian	286	138	125	23	401	0.70	171	0.30	0.47
9	Roy et al. (2018)	India (West)	Asian	270	132	115	23	379	0.70	161	0.30	0.77
10	Kaur et al. (2019)	India (North)	Asian	150	63	72	15	198	0.66	102	0.34	0.39
	Present study	India	Asian	400	118	220	62	456	0.57	344	0.43	0.014

The observed genotype frequencies among 314 male were: A2A2 = 97 (30.8%), A1A2 = 167 (53.1%), and A1A1 = 50 (15.9%). The allele frequencies were A2 = 0.57 and A1 = 0.43. The expected genotype frequencies under HWE were A2A2 = 103.75, A1A2 = 153.48 and A1A1 = 56.75. The male population is in HWE (p = 0.118). The genotype frequencies observed amongst 86 females were as follows: A2A2 = 21 (24.4%), A1A2 = 53 (61.6%), A1A1 = 12 (13.9%). The allele frequencies are A2 = 0.55 and A1 = 0.0.447. The expected frequencies under HWE are A2A2 = 26.23, A1A2 = 42.52 and A1A1 = 17.23. The population is deviated from HWE (p = 0.02) and F (is) = -0.247 again suggest heterozygous excess.

This deviation may be attributed to various factors such as regional/population admixture or stratification which is common in Indian populations as a consequence of caste mixing and migration (castelevel outbreeding rather than inbreeding), possible selective advantage for heterozygotes (Rudan et al., 2006; Li and Leal, 2009; Sen and Burmeister, 2008; Piel et al., 2016). Evidences suggest that both A1 and A2 allele confer advantages in different environmental or neurological context. Balancing selection could maintain both the alleles in the population leading to higher than expected heterozygosity. Potential selection pressure favoring heterozygosity at this locus is consistent with the findings of psychiatric genetics where intermediate dopaminergic receptor availability is beneficial for behavioral regulation (Hayden et al., 2010).

## Literature screening

Following the literature retrieval, utilizing the initial strategy, a total of 2,760 studies were obtained. Upon step by step screening, 2,619 non standard studies were eliminated and 136 studies were finally enrolled. A sum of 136 studies meets the inclusion criteria, the DRD2 Taq1 A polymorphism data involving the six continents of the world were covered. The frequency distribution among all 136 articles included in the present study was delineated by geographic regions, North America (The United States of America (USA), Mexico, Canada), South America (Brazil), Europe (Germany, France, Europe, Sweden, Finland, United Kingdom (UK), Russia, Norway, Italy, Czech, Spain, Türkiye, Netherland, Switzerland, Hungary, Bosnia and Herzegovina, Poland), Oceania (Australia), Asia (Japan, Korea, India, China, Taiwan, Iran, Pakistan) and Africa (Egypt).

The correlation of DRD2 Taq1A polymorphism with several associated psychiatric disorders has been a research hotspot. It is one of the most widely studied polymorphism of the DRD2 gene due to its association with the reward pathways. The T (A1) allele carriers have contributed to reduced expression and the density of dopamine receptors in the striatum. The change in the level of dopamine as a consequence of reduced dopamine receptor density affects the dopaminergic pathways and thus is implicated in the pathogenesis of several psychiatric and neurological disorders (Spitta et al., 2022; Kumar et al., 2024). DRD2 Taq1 A polymorphism is extensively investigated in different continents and countries, and a large diversity in the frequency of the T allele is observed among the various ethnic populations worldwide.

Limited studies have been reported from Eastern Uttar Pradesh, India, highlighting the need for localized research. Therefore, this study was designed, and the findings were compared with data obtained from the global population. Awareness of the frequency of this SNP is essential because it confers genetic susceptibility to various disorders. Several published articles have identified the A1 allele of the DRD2 Taq1A polymorphism as a potential risk factor for various psychiatric disorders (Cordeiro and Vallada, 2014; Cai et al., 2015; Sukasem et al., 2016; Roy et al., 2018; Alfimova et al., 2019; Chiang et al., 2020; Ohira et al., 2022). According to the reports of World Health Organization (WHO), in 2019, approximately 970 million people-about 1 in 8 individuals globally-were living with mental health disorders, with depression and anxiety being the most prevalent. Furthermore, after 2019, there has been a significant increase in the global burden of psychiatric disorders, primarily due to the effects of the COVID-19 pandemic (World Health Organization, 2022). The Global Burden of Diseases (GBD) showed that mental disorders are among the top ten leading causes of burden worldwide with no evidence of reduction in the global burden since 1990. The global number of disability-adjusted life years (DALYs) due to mental disorders has increased from 80.8 million to 125.3 million between 1990 and 2019 (GBD and Mental Disorders Collaborators, 2022). To reduce the burden of mental disorders, coordinated delivery of effective prevention and treatment programs by governments and the global health community is imperative. Hence in the present study, we aimed to compare the frequency obtained in the present study with the frequency reported worldwide.

**FIGURE 3:**
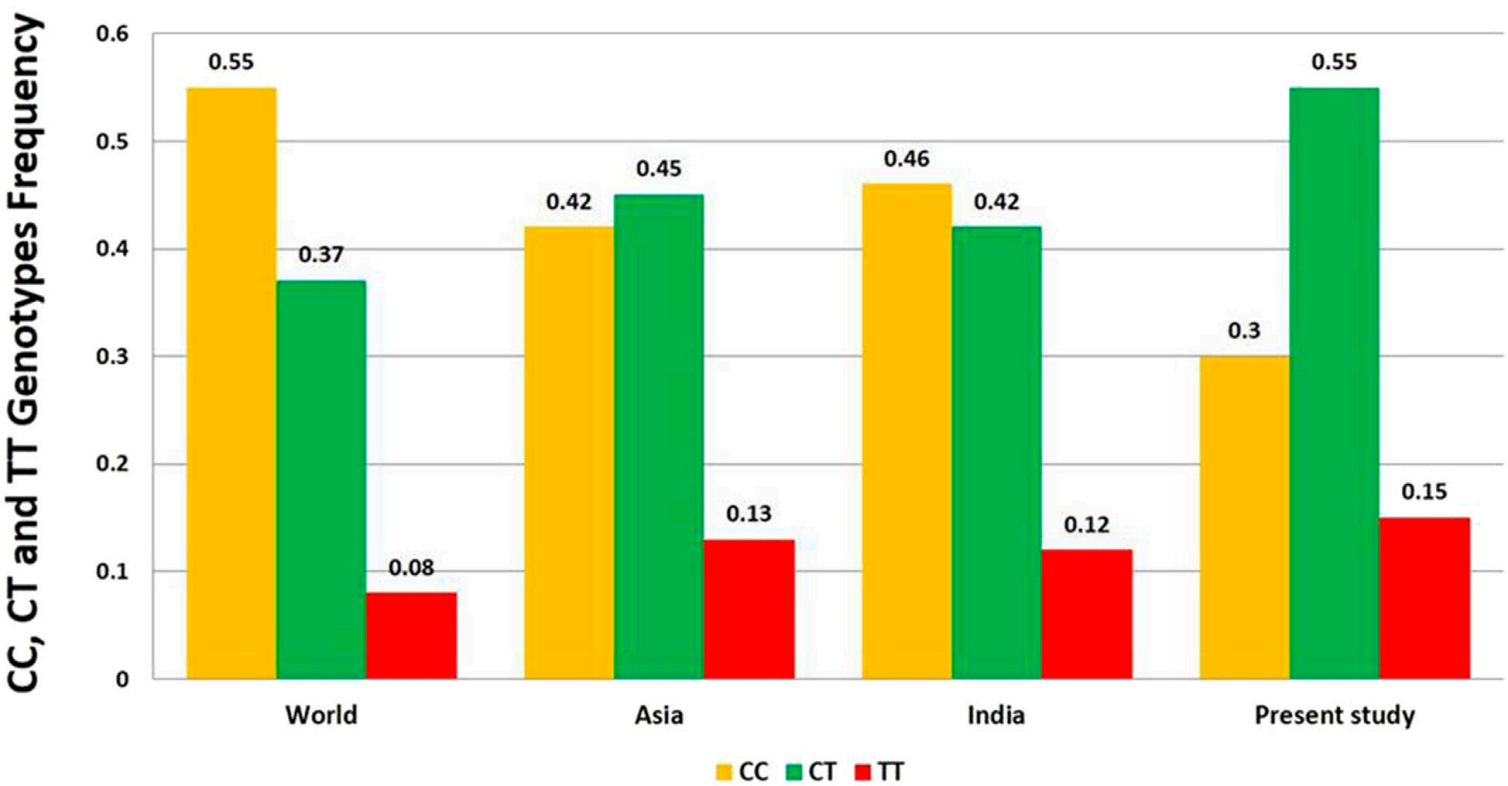
Distribution of CC, CT and TT genotypes frequency of DRD2 Taq1A polymorphism in World, Asia, India and present study.

Globally, out of the total control samples, the frequency of genotype CC, CT and TT was found to be 0.55 (55%), 0.37 (37%), and 0.08 (8%) respectively. The C and T allele frequencies were 0.74 and 0.26 respectively (Figure 3). Global frequency of T allele ranges from 0.06 to 0.61 (Parsian et al., 1991; Goldman et al., 1997).

In the North American continent, the T allele frequency of DRD2 Taq1A ranges from 0.06 (USA, Missouri, all subjects were white) (Parsian et al., 1991) to 0.61 (USA, South western American Indian population located in Arizona) (Goldman et al., 1997). The frequency of CC, CT, and TT genotypes was calculated as 0.58, 0.34 and 0.08 respectively and the frequency of C allele was 0.75, and T allele was 0.25. The wild type CC genotype was more frequent in the population of the North America, with the exception of the population belonging to Mexican ethnicity, where the heterozygous CT genotype prevails. Higher CT genotype combination increases the likelihood of higher T allele frequency in Mexicans. The T allele frequency observed in Mexicans, Mexican American and Indian American are 53%, 56%, and 61% respectively (Nicolini et al., 1996; Goldman et al., 1997; Konishi et al., 2004). The possible reasons for the remarkable difference in Mexicans can be explained by several genetic and population-specific factors such as population stratification, genetic relationships, and asymmetrical admixture or differential gene flow (Martinez-Cortes et al., 2012; Spear et al., 2020).

Mexicans are generally comprised of a combination of Native American and Spanish ancestry. Mexican Americans are estimated to have 31% Native American, 61% Spanish, and 8% African genes. This reflects the mixed genetic background of the Mexicans and Mexicans American population supporting the difference in the gene frequencies. (Konishi et al., 2004; Gómez et al., 2021; Gutiérrez-Virgen et al., 2023; Álvarez-Topete et al., 2024). Over generations, migrations, genetic relationships and admixture components might have increased the frequency of T allele (Martinez-Cortes et al., 2012). In other regions, T allele frequency ranges from 6% to 41% in USA, and 18% in Canada. Population of the North America had the total frequencies of 8% and 25% in the TT genotype and T allele respectively.

In South America, the T allele frequency of DRD2 Taq1A varies from 0.17 (European derived population from South Brazil, mainly of Portuguese descent along with the contribution of Spaniards and Germans) (Freire et al., 2006) to 0.41 (Brazil) (Cordeiro and Vallada, 2014). The distribution of the frequency of genotype CC, CT and TT was observed 0.46 (46%), 0.42 (42%), and 0.12 (12%) respectively. The C allele, and T allele frequencies were as 0.67, and 0.33 respectively. In South American population from Brazil, CC genotype was also observed to be highest except in a single study where CT genotype is more frequent. This difference is possibly due to the ethnical stratification in the population of highly admixed ethnicity like Brazilian one (Cordeiro and Vallada, 2014). The total frequency of TT genotype is 12% and T allele is 33% in the population of South America.

In European continent, the T allele frequency ranges from 0.08 (South East and North West of France) (Amadeo et al., 1993) to 0.49 (Ankara province of Turkiye) (Aslan et al., 2010). The frequency of the CC, CT, and TT genotypes was observed 0.65 (65%), 0.31 (31%) and 0.04 (4%), respectively. The frequencies of C and T alleles in European were 0.81, and 0.19 respectively. The wild type CC leads in the populations of the European continent reflecting higher frequency of C allele. However, in specific region of France, UK, Finland and Turkiye, some studies have reported higher frequency of CT (heterozygous) genotype (Li et al., 2002; Dubertret et al., 2004; Nyman et al., 2007; Aslan et al., 2010). Europe is not genetically uniform, for instance, Southern and Eastern Europe (such as Italy, Greece, Turkiye) might display more genetic admixture due to historical migrations (for example, Turkiye exhibit considerable genetic admixture with Middle Eastern and central Asian linage), in contrast, Northern Europe (Such as UK, Finland) might have distinct founder effects or drift patterns (France and UK have experienced historical gene flow from Celtic, Roman, Nordic and other populations) (Pinhasi et al., 2012; Lazaridis et al., 2014). Amongst the total subjects of European continent, the TT genotype frequency was 4% and the T allele frequency was 19%. The T allele frequency in France (46%) (Amadeo et al., 2000), Finland (46%) (Nyman et al., 2007) and Turkiye (49%) (Aslan et al., 2010) ranked among the top three in the European continent. However, in these regions, a higher range of variation is observed. There is a gradual increase in the T allele frequency in the population from 8% to 46% in France (Amadeo et al., 1993; 2000), 11%–46% in Finland (Hietala et al., 1997; Nyman et al., 2007), 16%–49% in Turkiye (Aslan et al., 2010; Nacak et al., 2012).

DRD2 Taq1 A polymorphism manifests notable variations in genotype and allele frequencies across populations of different ancestries. Mexicans and most of the South Americans are broadly categorized as Latinos or Hispanics, but despite this, genetic differences exist due to their distinct ancestral composition, that influences the distribution pattern of the DRD2 Taq1 A polymorphism. There is higher T allele frequencies and CT/TT genotypes in Native- American-rich population (such as Mexico), and higher C allele frequencies and CC genotypes in Europe, Australia, Africa and North America. In Europe, the C allele is more frequent. In contrast, European derived populations in Canada and United States displays comparable overall pattern with subtle difference. Amongst non-Hispanic White Americans and Canadians of European descent, the genotype frequencies mirrors to those of Europe.

The CC genotype remains more frequent in African population (Egypt) (55%) (Arab and Elhawary, 2015), consistent with Europe and European derived population like Canada and USA. However, the T allele frequency in Africa is slightly higher (0.29) than Europe (0.19) and comparable to North America (0.25). The frequency of TT genotype and T allele is 12% and 29% respectively. The Australian population shows higher CC genotype frequency (65.3%) and low TT genotype frequency (3.9%) and highest C allele frequency (80.7%) amongst all regions. This suggests a strong European ancestral influence, since Australian population is largely of European descents (especially from the UK and Ireland). The TT genotype accounts for 2% (South Australia) (Barratt et al., 2006) to 6% (Australians of Caucasian descent) (Lawford et al., 1997) while, the T allele from 17% (Asutralia) (Voisey et al., 2009) to 28% (Australia) (Oei et al., 2012). In the Asian population, the range of frequency of the T allele of DRD2 Taq1A gene accounts for 0.09 (Iran) (Najafabadi et al., 2005) to 0.52 (Taiwan) (Lee et al., 2005). The observed frequency of the genotype CC, CT, and TT was 0.42 (42%), 0.45 (45%), and 0.13 (13%), respectively. The frequency of C allele was 0.65 and T allele was 0.35. The highest T allele frequency was observed in the China (26%–52%) (Li et al., 2002; Lee et al., 2005).

After retrieval and comparison with the data of all other continents it was observed that the heterozygous CT type is more frequent in the most of the Asian population studied unlike other populations of North and South America, Africa, Europe and Australia, where CC predominates. The overall T allele frequency in Asia is 35% which is highest amongst all except Mexico. This represents significant population level diversity within Asia due to admixture of East Asia, South Asia and South East Asian ancestry and ethnic diversity.

A total of ten case-control studies were published from India (Shaikh et al., 2001; Juyal et al., 2006; Vijayan et al., 2007; Singh et al., 2008; Bhaskar et al., 2010; Prasad et al., 2010; Kumudini et al., 2013; Singh et al., 2013; Roy et al., 2018; Kaur et al., 2019). T allele frequency in control group of all the ten studies ranges from 0.22 (North Indian) (Prasad et al., 2010) to 0.47 (South Indian) (Bhaskar et al., 2010). The distribution of frequency of the genotype CC, CT, and TT in India was 0.46 (46%), 0.42 (42%), and 0.12 (12%) respectively. The frequency of C allele was 0.67 and T allele was 0.33. The T allele frequency in India (33%) is moderately high when compared to Europe and Australia (19%) and is comparable to Asia (35%). These results display high genetic diversity which is likely be influenced by Ethnolinguistic and caste based subdivisions, population admixture between Indo-European, Dravidian and Astroasiatic ancestries (Sinha et al., 2015; Kaur et al., 2019). In the present study, the genotype frequency of CC, CT and TT obtained was 0.30 (30%), 0.55 (55%) and 0.15 (15%). The C and T alleles were 0.57 and 0.43 respectively, which were comparable to previously published Indian and Asian studies. The CT genotype is most common indicating high heterozygosity in the population. The C allele is still more frequent than T allele. However, T allele frequency is relatively higher compared to other Indian and global populations (surpassed only by the Mexican and Mexican-American populations). The genotypic pattern in the present study is quite close to Mexicans. This suggests high genetic diversity, possibly subpopulation specific influences such as endogamy, caste variation or founder effects (Sinha et al., 2015). This result supports the need to interpret dopaminergic gene variants within regional population contexts instead of deducing from pan-Indian or Global averages.

**FIGURE 4:**
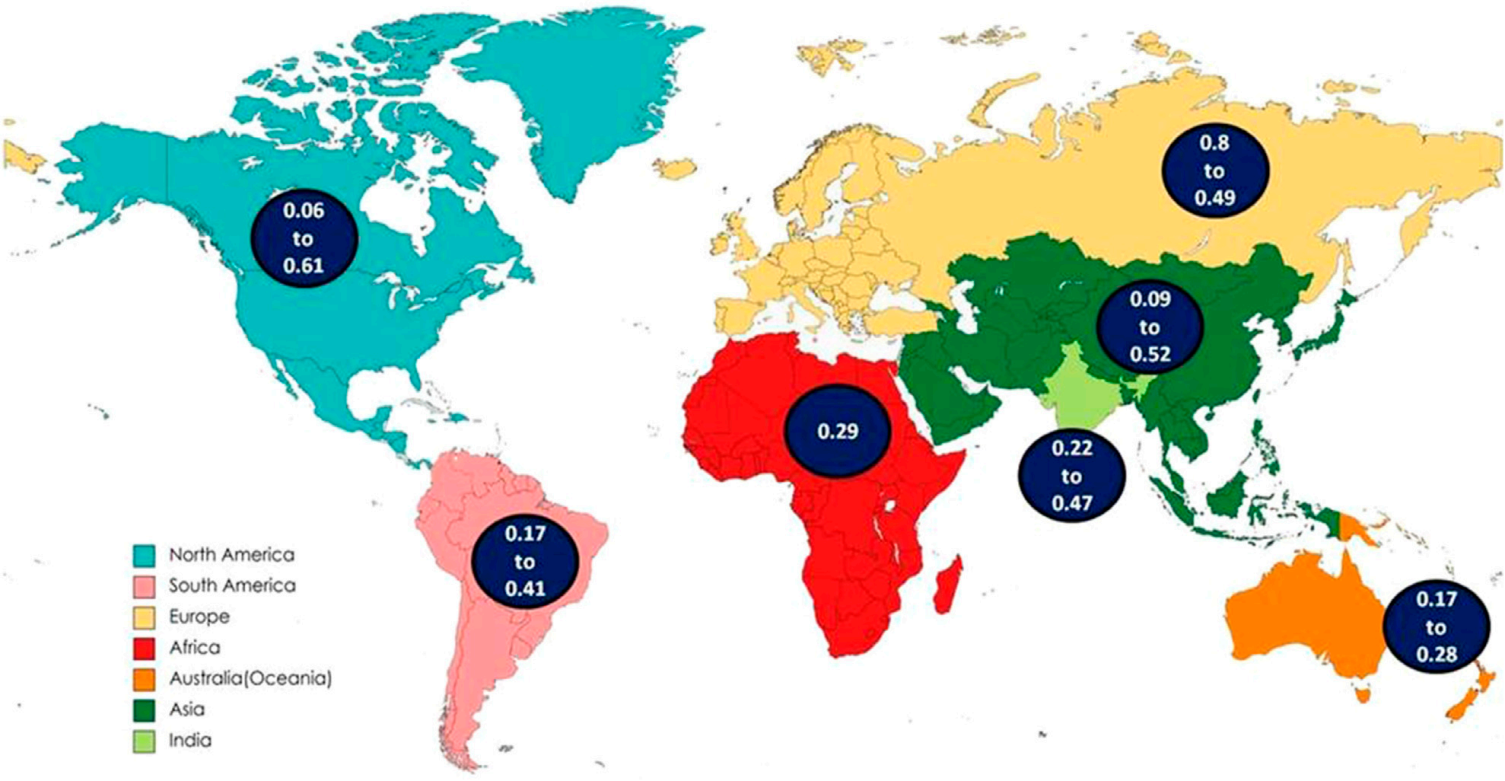
Map showing global distribution of T allele.

The interpretation of Global pattern suggest, the highest T allele frequency in Asia (0.35), moderate frequencies in South America (0.33) and Africa (0.29), Lowest frequencies in Europe and Australia (0.19) and intermediate frequency in North America (0.25). In the present study, the frequency of T allele was found to be 43% in India (15%, TT genotype) which is in concurrence with the reports of the Asian continent (Figure 4).

Along with these, some of the limitations must be acknowledged to ensure the validity of the findings, such as this study is based on the single regional population, which may not represent broader Indian diversity. This study does not include behavioural, neurological or clinical data for its association with genotype. To address population stratification, larger and more diverse samples from different regions of India must be warrant.

In conclusion, the results of DRD2 Taq1Apolymorphism analysis showed the T allele frequency as 43% which is quite high. As reported earlier, individuals with T allele tend to have lower dopamine receptor density, which might influence their response to reward-modulating therapies. So, T allele frequency determination helps in developing population-specific strategies, paving the ways for personalized treatment. Understanding the frequency distribution can guide screening programs in populations with high risk for psychiatric disorders especially AUD, and supports targeted awareness campaigns, early diagnosis, coordinated delivery of effective preventive strategies, and treatment programs by government and global health community to reduce the burden of disease.

## Data Availability

The datasets presented in this study can be found in online repositories. The names of the repository/repositories and accession number(s) can be found in the article/supplementary material.
